# Quantitative Analysis and Human Health Risk Assessment of Heavy Metals in Paddy Plants Collected from Perak, Malaysia

**DOI:** 10.3390/ijerph19020731

**Published:** 2022-01-10

**Authors:** Agatha Anak Sibuar, Nur Syahirah Zulkafflee, Jinap Selamat, Mohd Razi Ismail, Soo Yee Lee, Ahmad Faizal Abdull Razis

**Affiliations:** 1 Department of Food Science, Faculty of Food Science and Technology, Universiti Putra Malaysia (UPM), Serdang 43400, Selangor, Malaysia; agathasibuar@gmail.com (A.A.S.); nursyahirahzulkafflee@gmail.com (N.S.Z.); sjinap@gmail.com (J.S.); 2 Laboratory of Food Safety and Food Integrity, Institute of Tropical Agriculture and Food Security, Universiti Putra Malaysia (UPM), Serdang 43400, Selangor, Malaysia; 3 Laboratory of Climate-Smart Food Crop Production, Institute of Tropical Agriculture and Food Security, Universiti Putra Malaysia (UPM), Serdang 43400, Selangor, Malaysia; razi@upm.edu.my; 4 Natural Medicines and Products Research Laboratory, Institute of Bioscience, Universiti Putra Malaysia (UPM), Serdang 43400, Selangor, Malaysia; leesooyee@upm.edu.my

**Keywords:** rice, heavy metal, Perak, mining, human health risk

## Abstract

Rice is one of the major crops as well as the staple food in Malaysia. However, historical mining activity has raised a concern regarding heavy metal contamination in paddy plants, especially in Perak, a state with major tin mining during the late nineteenth century. Therefore, the objective of this study is to investigate the heavy metals (As, Cd, Pb, Cu, Cr) contamination in paddy soils and paddy plants in three districts in Perak. The content of heavy metals was determined using ICP-MS, while the absorption and transferability of heavy metals in the paddy plants were investigated through enrichment (EF) and translocation (TF) factors. Principal component analysis (PCA) was employed to recognize the pattern of heavy metal contaminations in different sampling areas. Health risk assessment was performed through calculation of various indices. The quantification results showed that root contained highest concentration of the studied heavy metals, with As exhibiting the highest concentration. The EF results revealed the accumulation of As, Cu, and Cr in the rice grains while PCA showed the different compositional pattern in the different sampling areas. The health risk assessment disclosed both noncarcinogenic and carcinogenic risks in the local adults and children. Overall, findings from this study show that heavy metal contamination poses potential health risks to the residents and control measure is required.

## 1. Introduction

Over the years, contamination of agricultural soil by heavy metals has become a critical pollution issue in developed and developing countries [1]. Besides being introduced naturally from parent rocks, the contamination of agricultural soil by heavy metals is caused by anthropogenic activities, such as industrialization, metal mining, application of unqualified fertilizer and pesticides, and wastewater irrigation [2]. The heavy metals commonly found in contaminated agricultural lands include lead (Pb), chromium (Cr), arsenic (As), zinc (Zn), cadmium (Cd), copper (Cu), mercury (Hg), and nickel (Ni) [3]. Unlike organic contaminants, these inorganic chemical hazards are not biodegradable and hence able to accumulate in the soil up to hazardous level [4]. Eventually, these contaminants can pose risk on human health via food consumption. In view of the huge consumption of rice across the globe, rice has become a significant dietary source of heavy metals, especially in Asia, which accounts for over 90% of global rice production and consumption [5,6,7].

Like many other Asian countries such as Indonesia, Thailand, Brunei, and Philippines, rice is the major staple food in Malaysia. Rice is taken twice a day by a majority of Malaysian adults, usually during lunch and dinner, with an average intake of 2.5 plates per day [8]. Approximately 75% of the local demand of rice is supplied by the paddy plantation in the country, while the rest is supplemented by rice imported from countries such as Thailand and Vietnam [9]. Kedah is the state with largest paddy plantation areas in Malaysia. In relation to heavy metals contamination, several studies have been performed on paddy soils and paddy plants collected from plantation areas in Kedah [10,11,12,13,14]. Besides Kedah, the plantation areas in other rice-producing states, including Sabah and Selangor were also studied for heavy metals contamination on paddy soils and rice [15,16,17]. However, there is a lack of data for the safety of rice regarding to heavy metals contamination in Perak, a state with major tin mining and production in Peninsula Malaysia during the late nineteenth century. This historical 100 years of mining activity can contribute to contamination of soil in agriculture land, which will subsequently affect the crop plantation, as mining has been reported as a significant source of heavy metal contaminants [1,18].

In view of the huge consumption of rice among Malaysians and the impact of historical metal mining on agricultural land, the objective of this study is to analyze the contents of heavy metals (As, Cd, Pb, Cu, Cr) in paddy soils and paddy plants in Perak. The accumulation of heavy metals in the edible part of paddy (rice grains) was assessed using enrichment factor (EF) while uptake and transferability of heavy metals in different parts of the plant was measured using translocation factors (TF). The compositional pattern of heavy metals in the soil and paddy plants collected from different plantation areas was also compared. In addition, the health risk with respect to daily consumption of rice for local adults and children was also assessed. Findings from the current study may provide information regarding heavy metal accumulation in rice cultivated in Perak and serve as a basis for comparison to other regions in Malaysia.

## 2. Materials and Methods

### 2.1. Study Areas and Sampling

The samples of paddy soil and paddy plants were collected from three different districts in Perak, namely Kerian, Perak Tengah and Hilir Perak (Figure S1). At each study areas, three plots (1000 m^2^ each) were randomly selected for sampling, where three samples of paddy soil and paddy plants were collected from each plot. For paddy soil samples, the soil was retrieved from 0–30 cm depth of the sampling area using a soil hand auger. Meanwhile, for paddy plant samples, the whole plants were uprooted with the soil. The samples were kept in clean plastic bags and labelled according to the sampling sites, which were P1K, P2K, and P3K for Kerian, P1PT, P2PT, and P3PT for Perak Tengah, and P1HP, P2HP, and P3HP for Hilir Perak.

### 2.2. Sample Preparation and Heavy Metals Extraction from Soil

Prior to extraction, the soil samples were oven dried (Memmert Oven, Schwabach, Germany) at 50 °C, followed by grinding and sieving through a 250 μm mesh sieve (No. 60 mesh sieve). To extract heavy metals from the soil samples, 1 M ammonium acetate (NH_4_CH_3_COO) at pH 7 was used [10,15]. Briefly, 10 g of soil were mixed with 50 mL of NH_4_CH_3_OO in a conical flask and the mixture was shaken for 1.5 h. After centrifuge at 3000 rpm for 30 min, the samples were filtered through a 0.45 µm syringe filter (Millipore, Merck, Kenilworth, NJ, USA).

### 2.3. Sample Preparation and Heavy Metals Extraction from Paddy Plants

The collected paddy plants were separated into four different parts, namely, grains, leaves, stem and roots. All parts of the paddy plants were cleaned with running tap water, followed by rinsing with deionized water. The samples were then dried in a drying oven (Memmert Oven, Schwabach, Germany) at 50 °C until constant weight was achieved. The dried paddy samples were ground into fine powder using a grinder machine and the powdered samples were sieved through a 250 μm mesh sieve (No. 60 mesh sieve). Heavy metals in different parts of the paddy plants were extracted using acid digestion method [10]. To 1 g of each sample powder, 10 mL of 69% nitric acid (HNO_3_) was added. The mixture was heated at 60–80 °C on a hot plate for the digestion to take place. 60% perchloric acid (5 mL) was added periodically, until a clear solution was obtained, indicating the completion of digestion process. The cooled sample solution was filtered through 0.45 µm syringe filter (Millipore, Merck, Kenilworth, NJ, USA) and finally diluted to 50 mL with distilled water in a volumetric flask.

### 2.4. Inductively Coupled Plasma-Mass Spectrometry (ICP-MS)

The content of heavy metals (As, Cd, Pb, Cu, Cr) in the digested samples of paddy soil and different parts of the paddy plants were analysed using inductively coupled plasma-mass spectrometry (ICP-MS) (Perkin Elmer Elan DRC-e, Santa Clara, CA, USA) in µg/kg unit. The instrument was calibrated with standard solution before any analysis being done with real samples. Prior to real samples analysis the calibration in standard solution must be passed with r^2^ = 0.999. For quality assurance, all the laboratory equipment were soaked with 10% HNO_3_. A standard solution was analysed after every 10 experimental samples, and each soil and rice sample were analysed in triplicate together with Certified Reference Material (CRM) IRMM 804 in the range of 90.5% to 102.4% [10].

#### Instrumentation

The ICP-MS was tuned with 1 µg/L tuning solution from the ICP-MS Stock Tuning Solution diluted with ultra-pure water. The instrument was checked daily, and the performance checking was done for ideal performance of the instrument. The operational condition of the instrument was revealed in Table 1.

### 2.5. Heavy Metal Absorption and Transferability

#### 2.5.1. Enrichment Factor (EF)

Enrichment factor (EF) is an index describing the mechanism of heavy metal absorption from the soil to edible part of paddy, which is the grains [19]. It is calculated as followed:

(1) $$\mathrm{EF}=\frac{\mathrm{Cp}}{\mathrm{Cs}}$$

where Cp and Cs are the heavy metal concentration in the grain and soil, respectively. The EF > 1 indicates the ability of the plant to accumulate the metals while EF < 1 indicates the plant absorbs but does not accumulate heavy metals.

#### 2.5.2. Translocation Factor (TF)

Translocation factor (TF) is an index indicating the transferability of heavy metals from soil to the plant tissues (root, stem and grain) [19]. It can be calculated as follows:

(2) $$\mathrm{TF}=\frac{\mathrm{Cq}}{\mathrm{Cr}}$$

where Cq is the heavy metal concentration in the different parts of plant and Cr is the heavy metal concentration in the soil, root, or stem. The higher the translocation factor value, the greater the mobility of heavy metals from one part to another. There were three TF calculated in this study, which were TF from soil to root (TF_soil_), root to stem (TF_root_), and stem to grain (TF_stem_).

### 2.6. Human Health Risk Assessment

The human health risk assessment of each contaminant is normally based on the estimation of the risk level and is classified as carcinogenic or non-carcinogenic health hazards. Thus, to estimate the heavy metal contamination and potential carcinogenic and non-cancer health risk through ingestion of heavy metals in rice, Hazard Quotients (HQ) was summed up to get hazard indices assuming that the metals share mechanisms of action.

#### 2.6.1. Average Daily Dose (ADD)

The average daily dose (ADD) of heavy metal contaminants via rice consumption was used to quantify the oral exposure dosage over a period of time, expressed as a daily dose per unit body weight (mg/kg/day). It was calculated as followed:

(3) $$\mathrm{ADD}=\frac{C\times \mathrm{IR}\times \mathrm{EF}\times \mathrm{ED}}{\mathrm{BW}\times \mathrm{AT}}$$

where C is the concentration of heavy metals in the rice (mg/kg), IR is the daily average consumption of rice in Perak (kg/day), Bw is the body weight (kg), EF is the exposure frequency (days/year). ED is the exposure duration (years, equivalent to the average lifespan). AT is average time (the product of EF and ED). The values of IR, EF, EF, and BW in relation to Malaysian adults and children were referring to a previous study [20] and are presented in Table S1.

#### 2.6.2. Noncarcinogenic Risk

To analyze the noncarcinogenic risk, the United States Environmental Protection Agency’s (USEPA’s) maximum acceptable oral dose for a toxic element was used as the reference dose (RfD) and hazard quotient (HQ) was calculated as followed [1]:

(4) $$\mathrm{HQ}=\frac{\mathrm{ADD}}{\mathrm{RfD}}$$

where ADD is average daily dose and RfD is reference dose. HQ < 1 reflects no potential noncarcinogenic risks while HQ > 1 reflects potential noncarcinogenic risks.

To assess the potential risk from the combined heavy metal exposure, the hazard index (HI), which is the sum of all HQs [20], was calculated:

(5) $$\mathrm{HI}=\sum^{\;}\mathrm{HQ}$$

Similar to HQ, HI < 1 reveals no potential noncarcinogenic risks while HI > 1 reveals the likelihood of noncarcinogenic risks.

#### 2.6.3. Carcinogenic Risk

To assess the carcinogenic risk, lifetime cancer risk (LCR), which is the incremental probability of human developing cancer over a lifetime, was calculated using formula as follow [1]:

(6) LCR=ADD×CSF

where ADD is the average daily dose (mg/kg/day) while the CSF is the cancer slope factor, taken from Integrated Risk Information System, USEPA [21].

Meanwhile, total cancer risk (CRt) was used to assess the effects of multiple carcinogenic elements, calculated by the summation of LCR [20]:

(7) $$\mathrm{CRt}=\sum^{\;}\mathrm{LCR}$$

The value of <10^−4^ is considered acceptable for both LCR and CRt, indicating no potential carcinogenic health risk. Although Cd and Cr have been classified as carcinogenic elements, there is no available data regarding their cancer slope factor (CSF) through ingestion pathway. Therefore, only As and Pb were considered for the assessment of carcinogenic health risks.

### 2.7. Statistical Analysis

For the heavy metal concentration in the paddy plants and soils, results were expressed as mean ± standard deviation of three replicates. One-way analysis of variance (ANOVA) with Tukey’s post hoc test was performed to determine the significance differences of heavy metal concentration among plots at a confidence level of 95%. Principal component analysis (PCA) was used to compare the pattern of heavy metal concentrations in the paddy soils and paddy plants collected from the three different sampling areas. The statistical analysis was performed using MS Excel (version 2013, Microsoft, Redmond, WA, USA) and Minitab (Version 18, Minitab Inc, State College, PA, USA) software.

## 3. Results and Discussion

### 3.1. Quantification of Heavy Metals in Different Parts of Paddy Plants

The concentration of heavy metals in the different parts (root, stem, leaf, and grain) of paddy plants collected from Kerian, Perak Tengah and Hilir Perak are presented in Table 2, Table 3 and Table 4, respectively. For the paddy plants collected from Kerian (Table 2), though there was slight difference in the results among the 3 plots, the concentration of As was the highest among the studied heavy metals in all the parts of the paddy plants, while Cd was the one with lowest concentration. The accumulation of heavy metals in the paddy plants in Perak Tengah (Table 3) showed the same trend as those collected from Kerian, in which As was the heavy metal with highest concentration in the all the parts of the paddy plants. However, while the Cd was the heavy metal contaminant with lowest concentration in root, leaf, and grain, the Pb in the stem was lowest in concentration as compared to other contaminants. Meanwhile, the paddy plants collected from Hilir Perak (Table 4) exhibited the same trend as those collected from Kerian for both heavy metals with the highest and lowest concentrations in all the plant parts, which was As as the most abundant and Cd as the least abundant ones.

Overall, the results showed that roots accumulated higher amount of heavy metals, as compared to other parts of the paddy plants. This could be due to the reason that roots play the role as a barrier during the translocation process of metals which will protect stem and grain from any contamination. Besides, the grains in the all the sampling areas showed a low level of heavy metals, suggesting that the plants had the capability to filter the heavy metals to prevent them from accumulate in the edible parts of the plants [22].

In addition, the results of this current study also showed that paddy plants collected from the plantation areas in Perak contained highest amount of As, as compared to other heavy metal contaminants. This could be attributed to the previous mining activity in Perak that led to the contamination of the agricultural land. This is in agreement with previous study that reported the elevated level of As in agricultural land in China due to the discharge of wastewater containing heavy metals from mining activity [1]. Apart from that, in the paddy plants collected from all the sampling areas, Cd was present with the lowest concentration among the studied heavy metals. This might be due to the reason that Cd is a metal that easily being absorbed by the crops and scatter readily to the other parts of the plants [17]. After all, the heavy metals in the paddy plants were still below the maximum permitted level stated in the Malaysian Food Regulation 1985 [23] and CODEX standard [24].

### 3.2. Quantification of Heavy Metals in Paddy Soils

The concentration of heavy metals in paddy soils collected from Kerian, Perak Tengah, and Hilir Perak are presented in Table 5. Among the studied heavy metals, the results showed that Cr and Pb were the most abundant heavy metal contaminant in the paddy soil, with the concentration ranged from 0.049–0.17 mg/kg and 0.0080–0.053 mg/kg, respectively. High concentration of the heavy metals in the paddy soil can be attributed to the application of the phosphate fertilizers by the farmers in the agricultural land. Since these fertilizers are manufactured from phosphate ore with high level of heavy metals such as chromium, lead, and iron, their usage will increase the levels of these heavy metals in the soils [25]. On the other hand, the paddy soils contained low content of Cu, with concentration ranged from 0.0018–0.039 mg/kg. Low bioavailability and dispersion of Cu in soil could be the factor of its low concentration in paddy soils [16]. The results of this current study were consistent with the previous study conducted on paddy soil collected from Papar, Sabah, where the soil contained high level of chromium and lead and low level of copper and cadmium [16]. However, all the heavy metals in the paddy soil collected from the three studied areas did not exceed the maximum allowable concentration, as stated in the Chinese Environmental Quality Standard for Soils, grade II (GB15618-2018) [26] and European standards agriculture soils [27].

### 3.3. Heavy Metal Absorption and Transferability

#### 3.3.1. Enrichment Factor (EF)

**Figure 1:**
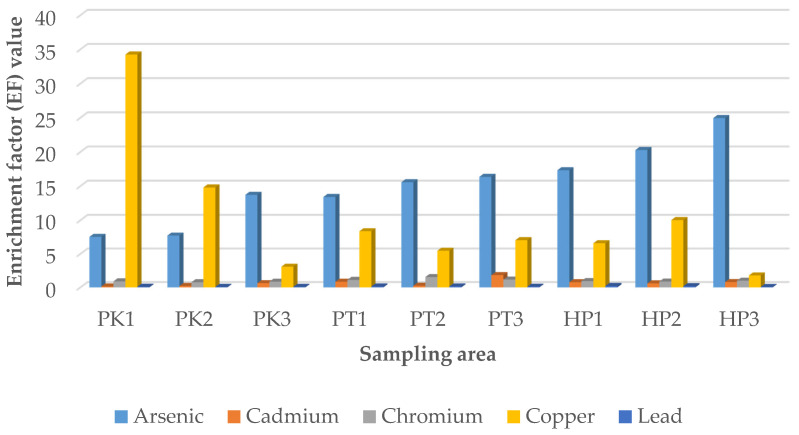
Enrichment factor (EF) from the soil to paddy grain of in the three studied areas. P1K, Plot 1 Kerian; P2K, Plot 2 Kerian; P3K, Plot 3 Kerian; P1PT; Plot 1 Perak Tengah; P2PT, Plot 2 Perak Tengah; P3PT, Plot 3 Perak Tengah; P3PT, Plot 3 Perak Tengah; P1HP, Plot 1 Hilir Perak; P2HP, Plot 2 Hilir Perak; P3HP, Plot 3 Hilir Perak.

Enrichment factor (EF) was calculated to evaluate the transfer of the studied heavy metals (As, Cd, Cr, Cu, Pb) from soils to rice grains and the results are displayed in Figure 1. The enrichment factor of the heavy metals was in decreasing order of As > Cu> Cr > Cd > Pb. Despite the low level of heavy metals in the grains as shown in the quantification results (Table 2, Table 3 and Table 4), there were three heavy metals in which the EF were recorded more than 1 based on the average reading, namely As, Cu, and Cr. According to the result, all plots in the three studied areas had EF more than 1 for As and Cu. The plots in Hilir Perak exhibited the highest EF for As, followed by the plots in Perak Tengah and Kerian, with the plots P3HP, P3PT, and P3K exhibited highest EF in each respective studied area (20.2727 for P2HP, 16.3556 for P3PT and 13.4091 for P3K). Meanwhile, for Cu, the plots P1K and P2K in Kerian showed a very high value of enrichment factor at 34.25 and 14.8, as compared to the other plot in the same studied area. This was because the concentration of Cu for grains in P1K and P2K plots were very high, possibly due to irrigation of wastewater water in these plots. In Perak Tengah and Hilir Perak, the highest reading of EF for Cu was at P1PT and P2HP, with value at 8.3718 and 10.0385, respectively. For Cr, the EF greater than 1 was only recorded in Perak Tengah and Hilir Perak. The highest enrichment factor was at the plot of P2PT with a reading of 1.5462 and P3HP with a reading of 1.0046 for both Perak Tengah and Hilir Perak. The enrichment factor greater than 1 implies that a plant does not only absorb the heavy metals, but also accumulate the heavy metals. The results of this study showed greater accumulation of As and Cu, followed by Cr, in the rice grains sampled from Kerian, Perak Tengah and Hilir Perak. This indicated that contamination of the agricultural soil by these heavy metals can pose potential human health risk via consumption of the rice grains [28].

#### 3.3.2. Translocation Factor (TF)

**Figure 2:**
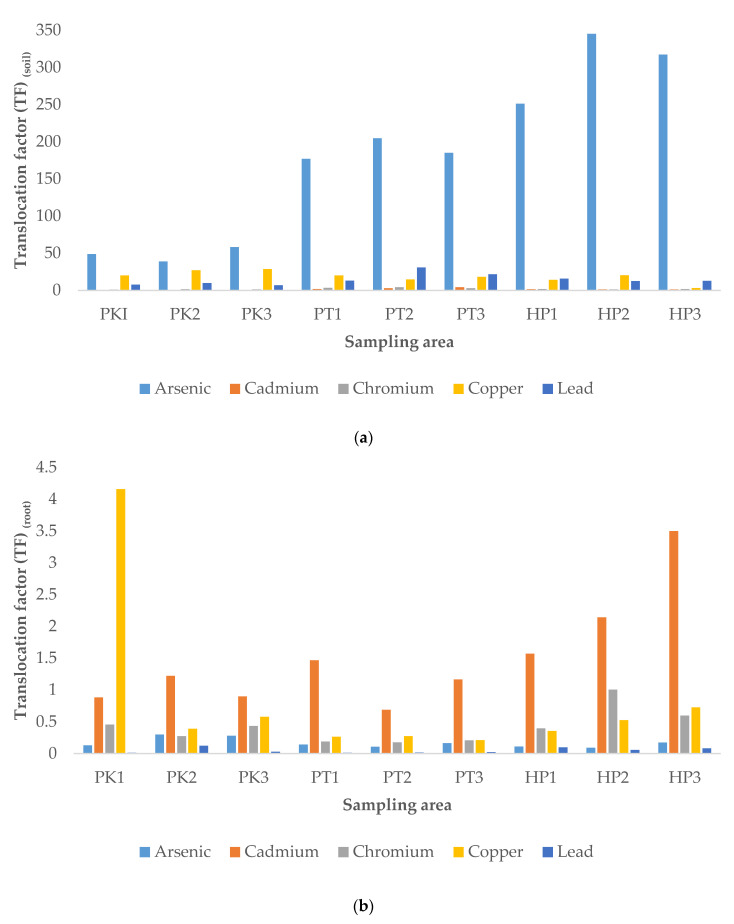

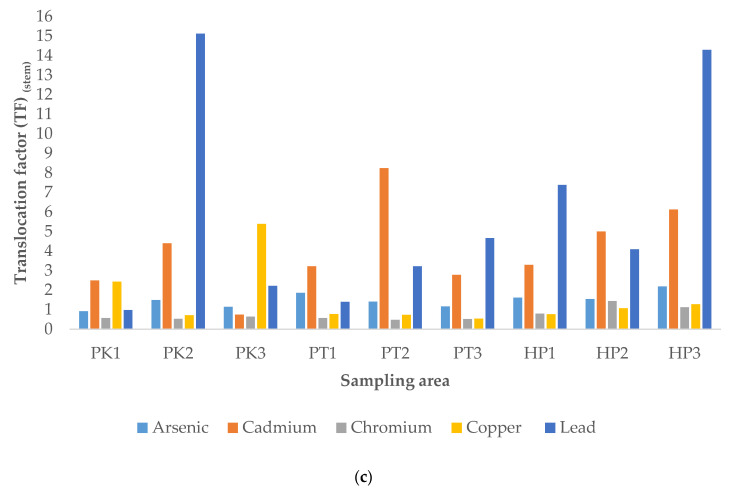
Translocation factor (TF) from soil to root (TFsoil, (**a**)), root to stem (TFroot, (**b**)) and stem to grain (TFstem, (**c**)). P1K, Plot 1 Kerian; P2K, Plot 2 Kerian; P3K, Plot 3 Kerian; P1PT; Plot 1 Perak Tengah; P2PT, Plot 2 Perak Tengah; P3PT, Plot 3 Perak Tengah; P3PT, Plot 3 Perak Tengah; P1HP, Plot 1 Hilir Perak; P2HP, Plot 2 Hilir Perak; P3HP, Plot 3 Hilir Perak.

Translocation factor (TF) is used to determine the accumulation and mobility of heavy metals in plants. The TF greater than 1 indicating the availability of heavy metals together with its mobility in the plants [29]. In this study, three EFs were calculated, which were EF from soil to root (TF_soil_), the root to stem (TF_root_) and stem to grain (TF_stem_), and the results are displayed in Figure 2. As shown in Figure 2a, the studied heavy metals in all the three studied areas exhibited TF_soil_ greater than 1, with the highest value exhibited by As (38.9286–345.4545). These results were in agreement with the quantification results discussed earlier, where the roots accumulated highest level of the As. For the TF_root_, the TF was in descending order of Cd > Cu > Cr > As > Pb. Among the studied heavy metals, only Cd showed TF_root_ greater than 1 in all the sampling plots, while only the sampling plot (PK1) revealed TF_root_ greater than 1 for Cu (Figure 2b). The TF_root_ of As, Cr, and PB were all less than 1. The high TF_root_ of Cd was owing to the high concentration of Cd in stem as compared to root, and this result was consistent with previous study [7]. Cd in nature has a high mobility in plants and hence can translocate more from root to stem [7]. For the TF_stem_, the TF was in the descending order of Pb > Cd > Cu > As > Cr (Figure 2c). All studied heavy metals exhibited the TF_stem_ beyond the acceptable level (>1) in all sampling areas except chromium. The Pb and Cd were among the heavy metals with highest TFs_tem_, which were in accordance with previous study, indicating that paddy plant was able to hyperaccumulate Cr and Pb from stem to grain [28]. The TF_stem_ value for Pb in this study was in range of 1.402 to 14.300, which was higher than the values (0.110–3.230) reported previously [16,30].

Overall, the results in this current study also revealed that the values of TF_soil_ for the studied heavy metals was greater than TF_root_ and TF_stem_, which were consistent with the results obtained by Singh et al. [31]. This indicates the heavy metals are more mobile from soil to the root of paddy plants, as compared to the mobility in other parts. This can be explained by the ability of the plants to tolerate the high concentration of heavy metals by restricting their transport from root to other parts by certain mechanism, such as accumulation in trichomes, exudates that can complex the heavy metals, linkages between the element and cell wall component, production of intracellular compounds with chelating properties and active pumping to the vacuoles [10].

### 3.4. Pattern Recognition of Heavy Metal Contamination in Different Sampling by PCA

**Figure 3:**
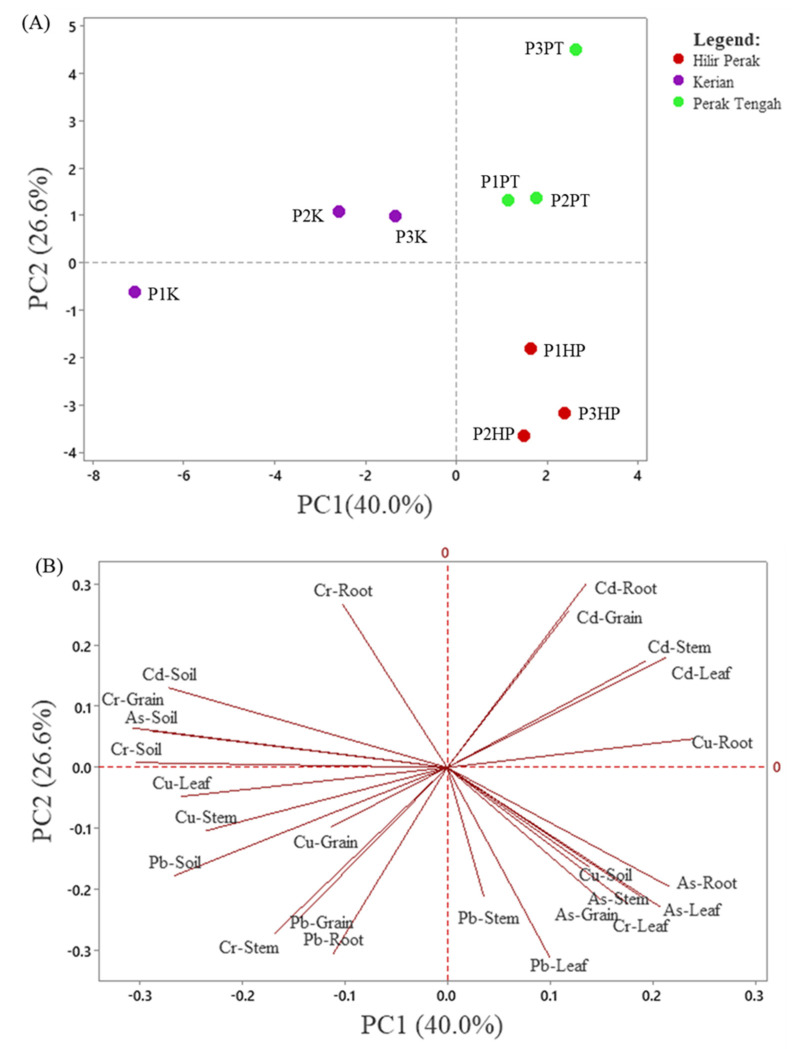
PCA score (**A**) and loading (**B**) plots of the different sampling areas. P1K, Plot 1 Kerian; P2K, Plot 2 Kerian; P3K, Plot 3 Kerian; P1PT; Plot 1 Perak Tengah; P2PT, Plot 2 Perak Tengah; P3PT, Plot 3 Perak Tengah; P3PT, Plot 3 Perak Tengah; P1HP, Plot 1 Hilir Perak; P2HP, Plot 2 Hilir Perak; P3HP, Plot 3 Hilir Perak.

Principal component analysis (PCA) was employed to compare the compositional pattern between the heavy metals in the soil and paddy plants collected from three different paddy plantation areas. PCA is a pattern recognition tool that can provide a primary understanding with regards to the similarity and dissimilarity between samples studied [32]. The PCA resulted in five principal components (PCs) with eigenvalues >1. Figure 3 shows the score and loading plots of the first two PCs, which have captured most of the variation from the data (66.6%). Score plot reveals the classification of the different sampling areas while the loading plot reveal the heavy metals in soil/paddy parts that contribute to the clustering characteristics of the sampling areas. The score plot (Figure 3A) shows that the sampling plots were well discriminated into three clusters, based on the paddy plantation areas. This shows that three studied paddy plantation areas are distinct from each other in relation to the contents of heavy metals in the soil and different parts of the paddy plants. The sampling plots in Perak Tengah and Hilir Perak were clustered at the positive side of PC1, with those in Perak Tengah (P1PT, P2PT, and P3PT) located at the upper quadrant while those in Hilir Perak (P1HP, P2HP, and P3HP) located at the lower quadrant, separated by the PC2. In contrast, the sampling plots in Kerian were clustered at the negative side of PC1. Within these plots, the P1K was discriminated from P2K and P3K by PC2, indicating there was intra-group variation in the sampling plots in Kerian.

From the loading plot (Figure 3B), it can be observed that the sampling plots in Kerian were discriminated by higher contents of Cr, Cd, As, and Pb in the soil, Cr and Pb in the root, Cr and Cu in the stem, Cu in the leaf, and Cr, Cu, and PB in the grain, with P2K and P3K contained higher contents of Cr, Cd, and As in the soil, and Cr in the root and grain, as compared to P1K. Meanwhile, the sampling plots in Perak Tengah (P1PT, P2PT, and P3PT) were characterized by higher contents on Cd in the paddy plants (root, stem, leaf, and grain), as well as the Cu in root. For the sampling plots in Hilir Perak, P1HP, P2HP, and P3HP were recognized by higher contents of Cu in soil, As in root, As, Cr and Pb in leaf, As and Pb in stem, and As in grain.

Overall, the PCA results revealed the sampling plots in Kerian has comparative higher level of heavy metal contamination in the soil. On the other hand, the sampling plots in Perak Tengah and Hilir Perak were characterized by higher accumulation of the heavy metals in the paddy plants, with relatively higher content of Cd in the plots in Perak Tengah and As in Hilir Perak sampling plots. The PCA results further support the EF results (Figure 1), which revealed the highest accumulation of As in the paddy grains collected from the plots in Hilir Perak. It is also noteworthy to mention that, among the studied heavy metals, As was the heavy metal with highest concentration in the paddy plants, as shown by the quantification results (Table 2, Table 3 and Table 4). Hence, consumption of the rice grains, especially those harvested from Hilir Perak can pose potential health risk to the local inhabitants.

### 3.5. Health Risk Assessment

The health risk assessment was defined on heavy metals exposure through the bioavailability of heavy metals in the paddy plants and paddy soil. The assessment composed of hazard quotient (HQ) and hazard index (HI) for non-carcinogenic risks and lifetime cancer risk (LCR) and total cancer risk (CRt) for carcinogenic risks of adults and children who are exposed to heavy metals through consumption of rice.

#### 3.5.1. Average Daily Dose (ADD)

One of the main routes of human exposure to the toxic heavy metals is through rice consumption that accumulated in the rice grain. Table 6 and Table 7 showed the average daily dose (ADD) of heavy metals among local adults and children through rice consumption. The ADD of As, Cd, Cr, Cu, and Pb through rice consumption were estimated to be 0.00081, 0.000011, 0.00074, 0.00048, 0.000029 mg/kg/day for adults and 0.00086, 0.000012, 0.00085, 0.00056, and 0.000031 mg/kg/day for children. The ADD for As, Cd and Cu for both adults and children were below the provisional tolerable daily intake obtained or calculated based on standards established by Joint FAO/WHO Expert Committee on Food Additives (JECFA) [33,34,35]. For Cr, there was insufficient toxicity studies to provide information regarding its no observed adverse effect level (NOAEL). This could be due to the difficulty in analyzing the hexavalent chromium separately as it was reduced to trivalent chromium in the stomach and gastrointestinal tract [36]. As for Pb, the previously established provisional tolerable weekly intake (PTWI) of 0.025 mg/kg body weight was withdrawn by JECFA in 2011, and a new and health-protective PTWI was not possible to be established, due to the reason of no clear indication of a limit for the major effects of this contaminant from the available dose–response analyses [35].

#### 3.5.2. Noncarcinogenic Risk Assessment

**Figure 4:**
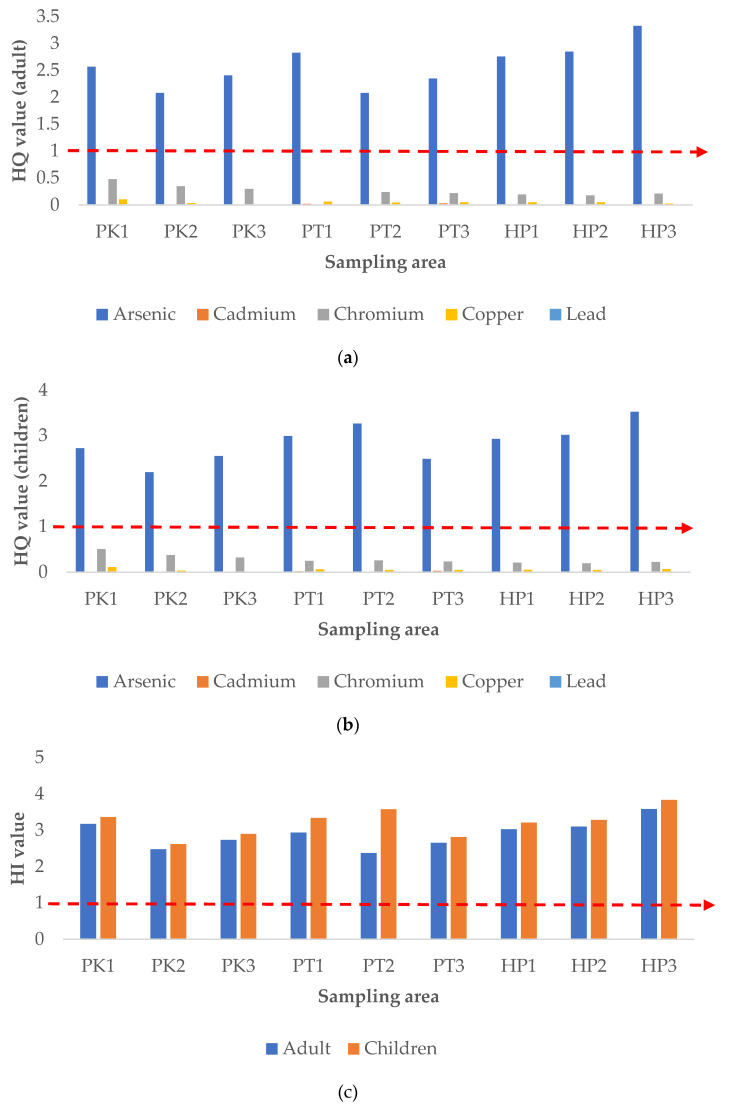
Hazard quotient (HQ, (**a**,**b**)) and hazard index (HI, (**c**)) of heavy metals for adult and children through rice consumption. P1K, Plot 1 Kerian; P2K, Plot 2 Kerian; P3K, Plot 3 Kerian; P1PT; lot 1 Perak Tengah; P2PT, Plot 2 Perak Tengah; P3PT, Plot 3 Perak Tengah; P3PT, Plot 3 Perak Tengah; P1HP, Plot 1 Hilir Perak; P2HP, Plot 2 Hilir Perak; P3HP, Plot 3 Hilir Perak.

The ADD was applied in the calculation of hazard quotient (HQ), which is an indicator to access the potential noncarcinogenic risk toward the human health. The results of HQ for the heavy metals through consumption of rice in Perak by adults and children were presented in Figure 4a,b. The trend of the results was similar for adults and children, with the descending order of As > Cr > Cu > Pb > Cd. The HQ for As was the highest and exceeded 1, which was in the ranges of 2.08 to 3.33 and 2.197 to 3.530 for adults and children, respectively. This suggested As poses potential noncarcinogenic risks for local people, both adults and children. Though the other heavy metals contaminants exhibited no obvious individual risk, the combined hazard index (HI) value for all the five studied heavy metals was beyond the acceptable value, which was more than 1 (Figure 4c). This indicated a potential non-carcinogenic risk to human health due to the combined exposure of these heavy metal contaminants in the rice. This result was similar to a previous study that reported that heavy metal contamination may pose potential health risk to local residents, especially those who are staying close to the mining areas [37]. Besides, the HI obtained from this study can likely be explained mainly by the As contamination, as its HQ value accounted for a huge proportion for the HI.

#### 3.5.3. Carcinogenic Risk Assessment

**Figure 5:**
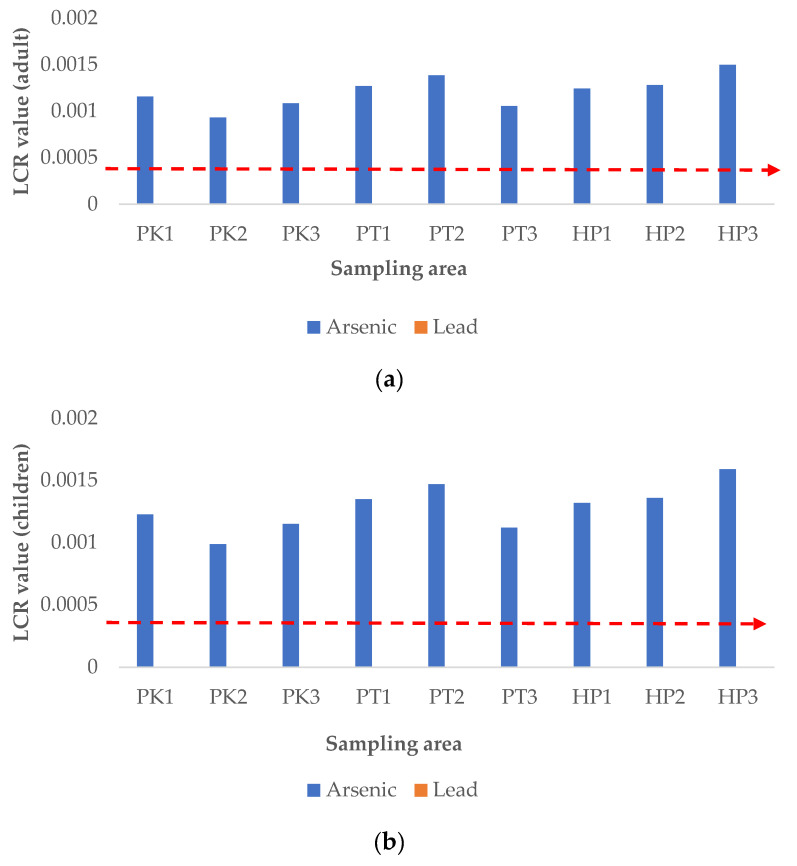

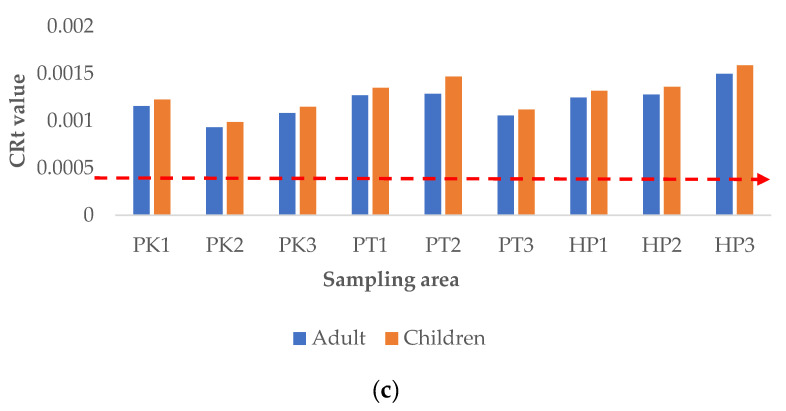
Lifetime cancer risk (LCR, (**a**,**b**)) and total cancer risk (CRt, (**c**)) of heavy metals for adults and children through rice consumption. P1K, Plot 1 Kerian; P2K, Plot 2 Kerian; P3K, Plot 3 Kerian; P1PT; lot 1 Perak Tengah; P2PT, Plot 2 Perak Tengah; P3PT, Plot 3 Perak Tengah; P3PT, Plot 3 Perak Tengah; P1HP, Plot 1 Hilir Perak; P2HP, Plot 2 Hilir Perak; P3HP, Plot 3 Hilir Perak.

The lifetime cancer risk (LCR) was calculated for As and Pb to assess their potential carcinogenic risk on adults and children through consumption of rice in Perak, and the results are shown in Figure 5. As recommended by USEPA, the acceptable LCR value is in the range of 10^−6^ to 10^−4^, which implies that one to one hundred in a million chance of additional human cancer over a 70-year lifetime is considered an acceptable risk [21]. The results of this study showed that the LCR for Pb was below 10^−4^, while the LCR for As exceeded the acceptable range, for both adults and children (Figure 5a,b). On the other hand, the total cancer risk (CRt), calculated from the summation of LCR of As and Pb, was beyond the acceptable limit, for both adults and children (Figure 5c). This revealed that there is a chance of getting cancer in the local inhabitants, both adults and children, through the combined exposure to As and Pb in rice. These results were supported by a previous study that showed increased carcinogenic risk resulted from consumption of rice contaminated by heavy metals from mining activities [1]. Notably, the CRt for the adults and children obtained in this study was contributed to the most by the As contamination.

## 4. Conclusions

This study evaluated the accumulation of heavy metals in paddy soil and paddy plants collected from three paddy plantation areas in Perak, a state with historical mining activity in Malaysia. The three studied paddy plantation areas were Kerian, Perak Tengah, and Hilir Perak. Overall, the quantification analysis using ICP-MS revealed that the roots accumulated a higher amount of heavy metals as compared to other parts of the paddy plants, with As as the most abundant one. Nevertheless, concentrations of these studied heavy metals in the paddy soils and paddy plants were still below the maximum permissible level stated in various standards, including Malaysian Food Regulation 1985, CODEX, Chinese Environmental Quality Standard for Soils, grade II (GB15618-2018) and European standards agriculture soils. Despite this, some of the heavy metals, especially As was found to be accumulated in the rice grains (EF > 1). The PCA results revealed the sampling plots in Kerian were characterized by a higher level of heavy metal contamination in the soil, while those in Perak Tengah and Hilir Perak were characterized by higher level of Cd and As, respectively in the paddy plants. Health risk assessment revealed the potential of both noncarcinogenic and carcinogenic risks, in both adults and children, in which the major concern felt on As. Both the calculated noncarcinogenic and carcinogenic risk factors of As exceeded the recommended standard values. The findings from this study suggested that historical mining activity in Perak is possible to cause non-cancer and cancer health effects due to heavy metal contamination of the agricultural land. Hence, regular monitoring of heavy metals in soils and rice in these areas, as well as other regions known for mining activities, is strongly recommended to ensure the safety of rice grain for consumption.

## Figures and Tables

**Table 1:** Operational condition of ICP-MS.

Items	Set Values
RF power	1200 W
Gas mode	He mode
Carrier gas	0.6–0.8 L/min
Dilution gas	0.40–0.60 L/min
Sampling depth	10.0–11.0 mm
Nebuliser	Miramist
Nebuliser gas flow	0.5 rps
Spray chamber	Quarts Scott style spray chamber (2 °C)
Interface cones	Platinum sampler and skimmer cones

**Table 2:** Concentration of heavy metals in paddy plants collected from Kerian.

Paddy Plant Parts	Plot	Concentration of Heavy Metals (mg/kg)				
		Arsenic (As)	Cadmium (Cd)	Chromium (Cr)	Copper (Cu)	Lead (Pb)
Root As > Pb > Cr > Cu > Cd	P1K	0.57 ± 0.0070 ^d^	0.0017 ± 0.000047 ^cd^	0.19 ± 0.013 ^ab^	0.064 ± 0.00078 ^ef^	0.40 ± 0.0046 ^a^
	P2K	0.33 ± 0.017 ^e^	0.0018 ± 0.00018 ^cd^	0.22 ± 0.040 ^a^	0.068 ± 0.0059 ^e^	0.30 ± 0.044 ^cd^
	P3K	0.32 ± 0.060 ^e^	0.0010 ± 0.000074 ^e^	0.14 ± 0.0021 ^bc^	0.052 ± 0.0038 ^f^	0.21 ± 0.014 ^e^
Stem As > Cu > Pb > Cr > Cd	P1K	0.075 ± 0.0012 ^c^	0.0015 ± 0.000094 ^ed^	0.086 ± 0.0040 ^a^	0.27 ± 0.023 ^a^	0.0051 ± 0.00045 ^cd^
	P2K	0.097 ± 0.0023 ^c^	0.0022 ± 0.000048 ^de^	0.060 ± 0.0022 ^bc^	0.027 ± 0.0003 ^d^	0.036 ± 0.048 ^c^
	P3K	0.090 ± 0.0014 ^c^	0.00090 ± 0.000015 ^f^	0.061 ± 0.0043 ^bcd^	0.030 ± 0.0010 ^d^	0.0060 ± 0.00043 ^cd^
Leaf As > Cu > Cr > Pb > Cd	P1K	0.13 ± 0.011 ^d^	0.00030 ± 0.000072 ^f^	0.015 ± 0.044 ^a^	1.6 ± 0.47 ^a^	0.011 ± 0.0011 ^ef^
	P2K	0.12 ± 0.015 ^d^	0.00080 ± 0.00012 ^de^	0.058 ± 0.0031 ^c^	0.043 ± 0.0077 ^b^	0.017 ± 0.0021 ^de^
	P3K	0.12 ± 0.0045 ^d^	0.00060 ± 0.000011 ^ef^	0.078 ± 0.018 ^bc^	0.062 ± 0.0032 ^b^	0.019 ± 0.00077 ^d^
Grain As > Cr > Cu > Pb > Cd	P1K	0.081 ± 0.0051 ^cde^	0.00060 ± 0.000050 ^bc^	0.15 ± 0.0036 ^a^	0.11 ± 0.0023 ^a^	0.0052 ± 0.0040 ^a^
	P2K	0.065 ± 0.0057 ^e^	0.00050 ± 0.00008 ^c^	0.11 ± 0.0040 ^b^	0.037 ± 0.0060 ^d^	0.0024 ± 0.0004 ^a^
	P3K	0.076 ± 0.0050 ^cde^	0.0012 ± 0.0016 ^bc^	0.094 ± 0.011 ^c^	0.0056 ± 0.012 ^bc^	0.0027 ± 0.00060 ^a^
Food Regulation 1985 ^A^		1.0	1.0	-	-	2.0
CODEX Standard ^B^		1.4	0.4	1.0	30	0.20

^a–f^ Value in a column with different superscripts refer to significantly different at *p* < 0.05. ^A^ The maximum permitted level of heavy metals in rice grain by Malaysian Food Regulation 1985; ^B^ The maximum permitted level of heavy metals in rice grain by CODEX standard. The data was collected from P1K (Plot 1 Kerian), P2K (Plot 2 Kerian), and P3K (Plot 3 Kerian).

**Table 3:** Concentration of heavy metals in paddy plants collected from Perak Tengah.

Paddy Plant Parts	Plot	Concentration of Heavy Metals (mg/kg)				
		Arsenic (As)	Cadmium (Cd)	Chromium (Cr)	Copper (Cu)	Lead (Pb)
Root As > Pb > Cr > Cu > Cd	P1PT	1.2 ± 0.088 ^b^	0.0048 ± 0.00029 ^b^	0.22 ± 0.013 ^a^	0.16 ± 0.0082 ^a^	0.31 ± 0.019 ^bc^
	P2PT	1.3 ± 0.12 ^b^	0.0048 ± 0.00027 ^b^	0.21 ± 0.027 ^a^	0.14 ± 0.0056 ^b^	0.26± 0.012 ^d^
	P3PT	0.83 ± 0.028 ^c^	0.0079 ± 0.00021 ^a^	0.17 ± 0.0047 ^abc^	0.13 ±0.0031 ^b^	0.18 ±0.0041 ^e^
Stem As > Cu > Cr > Cd > Pb	P1PT	0.16 ± 0.013 ^b^	0.0071 ± 0.00069 ^b^	0.042 ± 0.0036 ^de^	0.051 ± 0.0043 ^cd^	0.0046 ± 0.00020 ^cd^
	P2PT	0.14 ± 0.026 ^b^	0.0033 ± 0.00079 ^d^	0.036 ± 0.0051 ^e^	0.037 ± 0.0072 ^cd^	0.0039 ± 0.00097 ^d^
	P3PT	0.086 ± 0.0086 ^c^	0.0092 ± 0.00017 ^a^	0.039± 0.0027 ^e^	0.028 ± 0.00037 ^d^	0.0042 ± 0.0014 ^d^
Leaf As > Cu > Cr > Pb > Cd	P1PT	0.27 ± 0.0023 ^bc^	0.0010 ± 0.000018 ^cd^	0.053 ± 0.0054 ^c^	0.060 ±0.0048 ^b^	0.0085 ± 0.00026 ^f^
	P2PT	0.24 ± 0.016 ^c^	0.0015 ± 0.000083 ^b^	0.047 ± 0.0028 ^c^	0.082 ±0.0081 ^b^	0.011 ±0.00025 ^ef^
	P3PT	0.15 ± 0.0093 ^d^	0.0033 ± 0.0016 ^a^	0.052 ± 0.0022 ^c^	0.073 ± 0.013 ^b^	0.0084 ± 0.0012 ^f^
Grain As > Cr > Cu > Pb > Cd	P1PT	0.089 ± 0.0038 ^bcd^	0.0022 ± 0.00014 ^ab^	0.073 ± 0.0053 ^d^	0.065 ± 0.0064 ^bc^	0.0033 ± 0.0014 ^a^
	P2PT	0.097 ± 0.0014 ^ab^	0.00040 ± 0.000043 ^c^	0.075 ± 0.0020 ^d^	0.050 ± 0.0014 ^cd^	0.0012 ± 0.000034 ^a^
	P3PT	0.074 ± 0.0050 ^de^	0.0033 ± 0.00016 ^a^	0.069 ± 0.0044 ^d^	0.051 ± 0.0032 ^cd^	0.00090 ± 0.000036 ^a^
Food Regulation 1985 ^A^		1.0	1.0	-	-	2.0
CODEX standard ^B^		1.4	0.40	1.0	30	0.20

^a–f^ Value in a column with different superscripts refer to significantly different at *p* < 0.05. ^A^ The maximum permitted level of heavy metals in rice grain by Malaysian Food Regulation 1985; ^B^ The maximum permitted level of heavy metals in rice grain by CODEX standard. The data was collected from P1PT (Plot 1 Perak Tengah), P2PT (Plot 2 Perak Tengah), and P3PT (Plot 3 Perak Tengah).

**Table 4:** Concentration of heavy metals in paddy plants collected from Hilir Perak.

Paddy Plant Part	Plot	Concentration of Heavy Metals (mg/kg)				
		Arsenic (As)	Cadmium (Cd)	Chromium (Cr)	Copper (Cu)	Lead (Pb)
Root As > Pb > Cu > Cr > Cd	P1HP	1.3 ± 0.016 ^b^	0.0021 ± 0.000075 ^c^	0.12 ± 0.0040 ^cd^	0.12 ± 0.0029 ^cd^	0.34 ± 0.0021 ^abc^
	P2HP	1.5 ± 0.12 ^a^	0.0014 ± 0.00016 ^de^	0.082 ± 0.0088 ^d^	0.11 ± 0.0036 ^d^	0.36 ± 0.011 ^ab^
	P3HP	1.3 ± 0.082 ^ab^	0.0014 ± 0.000097 ^de^	0.12 ± 0.0098 ^cd^	0.12 ± 0.0072 ^bc^	0.35 ± 0.021 ^ab^
Stem As > Cr > Cu > Pb > Cd	P1HP	0.14 ± 0.0014 ^b^	0.0033 ± 0.00032 ^d^	0.049 ± 0.0032 ^cde^	0.041 ± 0.0044 ^cd^	0.034 ± 0.045 ^c^
	P2HP	0.14 ± 0.012 ^b^	0.0030 ± 0.00034 ^d^	0.082 ± 0.013 ^a^	0.056 ± 0.0072 ^c^	0.021 ± 0.0033 ^b^
	P3HP	0.23 ± 0.021 ^a^	0.0049 ± 0.00041 ^c^	0.074 ± 0.0098 ^ab^	0.089 ± 0.0071 ^b^	0.029 ± 0.031 ^a^
Leaf As > Pb > Cu > Cr > Cd	P1HP	0.30 ± 0.022 ^ab^	0.0015 ± 0.00012 ^b^	0.088 ± 0.0036 ^bc^	0.09 ± 0.068 ^b^	0.050 ± 0.045 ^b^
	P2HP	0.27 ± 0.013 ^bc^	0.0013 ± 0.00011 ^bc^	0.12 ± 0.017 ^ab^	0.12± 0.0012 ^b^	0.087 ± 0.0046 ^a^
	P3HP	0.33 ± 0.035 ^a^	0.0012 ± 0.000087 ^bc^	0.078 ± 0.015 ^bc^	0.12 ± 0.0034 ^b^	0.041 ± 0.0016 ^c^
Grain As > Cr > Cu > Pb > Cd	P1HP	0.087 ± 0.0052 ^bcd^	0.0010 ± 0.00035 ^bc^	0.061 ± 0.0029 ^d^	0.054 ± 0.0010 ^bcd^	0.0046 ± 0.0029 ^a^
	P2HP	0.089 ± 0.0069 ^bc^	0.00060 ± 0.000050 ^c^	0.057 ± 0.012 ^d^	0.052 ± 0.0043 ^cd^	0.0051 ± 0.00040 ^a^
	P3HP	0.10 ± 0.0091 ^a^	0.00080 ± 0.000073 ^bc^	0.066 ± 0.0049 ^d^	0.069 ± 0.0065 ^b^	0.002 ± 0.00053 ^a^
Food Regulation 1985 ^A^		1.0	1.0	-	-	2.0
CODEX standard ^B^		1.4	0.40	1.0	30	0.20

^a–e^ Value in a column with different superscripts refer to significantly different at *p* < 0.05. ^A^ The maximum permitted level of heavy metals in rice grain by Malaysian Food Regulation 1985; ^B^ The maximum permitted level of heavy metals in rice grain by CODEX standard. The data was collected from P1HP (Plot 1 Hilir Perak), P2HP (Plot 2 Hilir Perak), and P3HP (Plot 3 Hilir Perak).

**Table 5:** Concentration of heavy metals in paddy soil collected from all sampling areas.

Area of Paddy Field	Mean Concentration of Paddy Soil (mg/kg)				
	Arsenic (As)	Cadmium (Cd)	Chromium (Cr)	Copper (Cu)	Lead (Pb)
P1K	0.011 ± 0.0010 ^a^	0.0040 ± 0.00030 ^a^	0.17 ± 0.0075 ^a^	0.0032 ± 0.00056 ^b^	0.053 ± 0.0067 ^a^
P2K	0.0084 ± 0.00074 ^b^	0.0024 ± 0.00014 ^bc^	0.14 ±0.0048 ^a^	0.0025 ± 0.00021 ^b^	0.030 ± 0.00099 ^b^
P3K	0.0055 ± 0.0010 ^cde^	0.0019 ± 0.00052 ^bcd^	0.11 ± 0.021 ^b^	0.0018 ± 0.00038 ^b^	0.029 ± 0.0046 ^b^
P1PT	0.0066 ± 0.00032 ^c^	0.0026 ± 0.00047 ^b^	0.065 ± 0.0078 ^c^	0.0078 ± 0.0012 ^b^	0.024 ± 0.0051 ^b^
P2PT	0.0062 ± 0.00012 ^cd^	0.0016 ± 0.000078 ^cd^	0.049 ± 0.0014 ^c^	0.0092 ± 0.00061 ^b^	0.0085 ± 0.00079 ^c^
P3PT	0.0045 ± 0.00089 ^de^	0.0018 ± 0.00054 ^bcd^	0.059 ± 0.011 ^c^	0.0073 ± 0.0020 ^b^	0.0080 ± 0.0011 ^c^
P1HP	0.0050 ± 0.00014 ^cde^	0.0013 ± 0.000086 ^d^	0.065 ± 0.00033 ^c^	0.0082 ± 0.000077 ^b^	0.022 ± 0.0014 b
P2HP	0.0044 ± 0.00015 ^e^	0.0010 ± 0.000020 ^d^	0.065 ± 0.00017 ^c^	0.0052 ± 0.00019 ^b^	0.029 ± 0.0015 ^b^
P3HP	0.0042 ± 0.000032 ^e^	0.00098 ± 0.0000096 ^d^	0.066 ± 0.00068 ^c^	0.039 ± 0.021 ^a^	0.028 ± 0.00078 ^b^
GB15618-1195 ^A^	25	0.30	300	-	300
EU Standards ^B^	-	3.0	-	140	300

^a–e^ Values in a column with different superscripts between plots of each area refer to significantly different at *p* < 0.05; ^A^ Maximum allowable concentration of heavy metals in soil, recommended by the Chinese Environmental Quality Standard for Soils, grade II (GB15618-1995); ^B^ European standards agriculture soils. The data was collected from P1K (Plot 1 Kerian), P2K (Plot 2 Kerian), P3K (Plot 3 Kerian), P1PT (Plot 1 Perak Tengah), P2PT (Plot 2 Perak Tengah), and P3PT (Plot 3 Perak Tengah), P1HP (Plot 1 Hilir Perak), P2HP (Plot 2 Hilir Perak), and P3HP (Plot 3 Hilir Perak).

**Table 6:** The average daily dose (ADD) for heavy metals by adults through consumption of rice from Perak.

Area of Paddy Field	Average Daily Dose (ADD, mg/kg/day)				
	Arsenic (As)	Cadmium (Cd)	Chromium (Cr)	Copper (Cu)	Lead (Pb)
P1K	0.00077	0.0000058	0.0014	0.0011	0.000050
P2K	0.00062	0.0000048	0.0011	0.00035	0.000023
P3K	0.00072	0.000012	0.00090	0.000053	0.000026
P1PT	0.00085	0.000021	0.000070	0.00063	0.000031
P2PT	0.00092	0.0000038	0.00072	0.00048	0.000012
P3PT	0.00071	0.000032	0.00066	0.00049	0.0000086
P1HP	0.00083	0.0000096	0.00058	0.00052	0.000044
P2HP	0.00085	0.0000058	0.00054	0.00050	0.000049
P3HP	0.0010	0.0000077	0.00063	0.00025	0.000019
PTDI ^A^ (mg/kg/day)	0.0021 ^B^	0.00083 ^C^	-	0.50 ^D^	-

^A^ Provisional tolerable daily intake. ^B^ The PTDI of As was calculated from a provisional tolerable weekly intake (PTWI) of 0.015 mg/kg body weight. ^C^ The PTDI of Cd was calculated from a provisional tolerable monthly intake (PTMI) of 0.025 mg/kg body weight based on a month of 30 days. ^D^ The PTDI of Cu was taken based on the provisional maximum tolerable daily intake (PMTDI). P1K, Plot 1 Kerian; P2K, Plot 2 Kerian; P3K, Plot 3 Kerian; P1PT; Plot 1 Perak Tengah; P2PT, Plot 2 Perak Tengah; P3PT, Plot 3 Perak Tengah; P3PT, Plot 3 Perak Tengah; P1HP, Plot 1 Hilir Perak; P2HP, Plot 2 Hilir Perak; P3HP, Plot 3 Hilir Perak.

**Table 7:** The average daily dose (ADD) for heavy metals by children through consumption of rice from Perak.

Area of Paddy Field	Average Daily Dose (ADD, mg/kg/day)				
	Arsenic (As)	Cadmium (Cd)	Chromium (Cr)	Copper (Cu)	Lead (Pb)
P1K	0.00082	0.0000061	0.0015	0.0011	0.000053
P2K	0.00066	0.0000051	0.0011	0.00038	0.000024
P3K	0.00077	0.000012	0.00095	0.000057	0.000027
P1PT	0.00090	0.000022	0.00074	0.00066	0.000033
P2PT	0.00098	0.0000041	0.00076	0.00051	0.000012
P3PT	0.00075	0.000034	0.00070	0.00052	0.0000091
P1HP	0.00088	0.000010	0.00062	0.00055	0.000047
P2HP	0.00091	0.0000061	0.00057	0.00053	0.000052
P3HP	0.0011	0.0000081	0.00067	0.00071	0.000020
PTDI ^A^ (mg/kg/day)	0.0021 ^B^	0.00083 ^C^	-	0.50 ^D^	-

^A^ Provisional tolerable daily intake. ^B^ The PTDI of As was calculated from a provisional tolerable weekly intake (PTWI) of 0.015 mg/kg body weight. ^C^ The PTDI of Cd was calculated from a provisional tolerable monthly intake (PTMI) of 0.025 mg/kg body weight based on a month of 30 days. ^D^ The PTDI of Cu was taken based on the provisional maximum tolerable daily intake (PMTDI). P1K, Plot 1 Kerian; P2K, Plot 2 Kerian; P3K, Plot 3 Kerian; P1PT; Plot 1 Perak Tengah; P2PT, Plot 2 Perak Tengah; P3PT, Plot 3 Perak Tengah; P3PT, Plot 3 Perak Tengah; P1HP, Plot 1 Hilir Perak; P2HP, Plot 2 Hilir Perak; P3HP, Plot 3 Hilir Perak.

## Data Availability

Data is contained within the article or supplementary material.

## Supplementary Materials

The following are available online at https://www.mdpi.com/article/10.3390/ijerph19020731/s1, Figure S1: Three study areas located in Perak, Malaysia, Table S1: Information used for calculation of average daily dose (ADD) for Malaysian adults and children based on previous study [20].
