# Supplementary material for: Quantitative Analysis and Human Health Risk Assessment of Heavy Metals in Paddy Plants Collected from Perak, Malaysia
Source: Int J Environ Res Public Health. 2022 Jan 10;19(2):731. doi: 10.3390/ijerph19020731 (PMC8775821; doi:10.3390/ijerph19020731)
Supplement: Supplementary file 1 [file ijerph-19-00731-s001.zip › ijerph-1386793-supplementary.pdf]

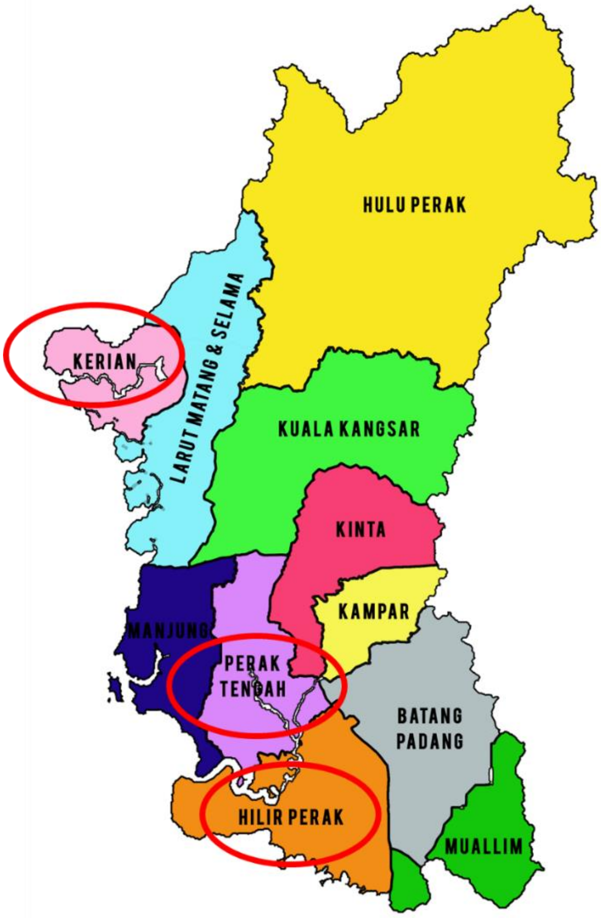

Figure S1. Three study areas located in Perak, Malaysia.

Table S1. Information used for calculation of average daily dose (ADD) for Malaysian adult and children based on previous study [20].

Parameters	Malaysian Adult	Malaysian Children
Ingestion Rate (IR, kg/day)	0.6	0.198
Exposure Frequency (EF, days/years)	365	365
Exposure Duration (ED, years)	74	74
Body Weight (BW, kg)	62.65	19.5
